# Improving performances of the knee replacement surgery process by applying DMAIC principles

**DOI:** 10.1111/jep.12810

**Published:** 2017-09-26

**Authors:** Giovanni Improta, Giovanni Balato, Maria Romano, Alfonso Maria Ponsiglione, Eliana Raiola, Mario Alessandro Russo, Patrizia Cuccaro, Liberatina Carmela Santillo, Mario Cesarelli

**Affiliations:** ^1^ Department of Chemical Engineering, Materials and Industrial Production University of Naples “Federico II” Naples Italy; ^2^ Department of Public Health University of Naples “Federico II” Naples Italy; ^3^ Department of Medical and Surgical Sciences University “Magna Graecia” of Catanzaro Catanzaro Italy; ^4^ University of Naples "Federico II" Naples Italy; ^5^ University Hospital “Federico II” Naples Italy; ^6^ S. Maugeri’ Foundation Scientific Institute of Telese Terme (BN), IRCCS Telese Terme Italy; ^7^ Department of Electrical and Information Technology Engineering University of Naples “Federico II” Naples Italy

**Keywords:** appropriate health care, delays and waiting lists, DMAIC cycle, length of hospital stay, quality management

## Abstract

**Rationale, Aims, and Objectives:**

The work is a part of a project about the application of the Lean Six Sigma to improve health care processes. A previously published work regarding the hip replacement surgery has shown promising results. Here, we propose an application of the DMAIC (Define, Measure, Analyse, Improve, and Control) cycle to improve quality and reduce costs related to the prosthetic knee replacement surgery by decreasing patients' length of hospital stay (LOS)

**Methods:**

The DMAIC cycle has been adopted to decrease the patients' LOS. The University Hospital “Federico II” of Naples, one of the most important university hospitals in Southern Italy, participated in this study. Data on 148 patients who underwent prosthetic knee replacement between 2010 and 2013 were used. Process mapping, statistical measures, brainstorming activities, and comparative analysis were performed to identify factors influencing LOS and improvement strategies.

**Results:**

The study allowed the identification of variables influencing the prolongation of the LOS and the implementation of corrective actions to improve the process of care. The adopted actions reduced the LOS by 42%, from a mean value of 14.2 to 8.3 days (standard deviation also decreased from 5.2 to 2.3 days).

**Conclusions:**

The DMAIC approach has proven to be a helpful strategy ensuring a significant decreasing of the LOS. Furthermore, through its implementation, a significant reduction of the average costs of hospital stay can be achieved. Such a versatile approach could be applied to improve a wide range of health care processes.

## 1 INTRODUCTION

Osteoarthritis (OA) is the most common disease of older population and one of the leading causes of disability. The definition of OA is not unique in reported studies and can include self‐reported OA obtained from questionnaires, radiographic definitions of OA, and symptomatic OA as defined by self‐reported joint pain and radiographic evidence.^1^ It has a major impact on health‐related quality of life compared with that of people without self‐reported musculoskeletal diseases. In many of the larger cohort studies, radiographic OA has been the preferred definition of incident OA.

Osteoarthritis of the knee is an active disease process involving cartilage destruction, subchondral bone thickening, and new bone formation. Incidence of knee OA is likely to grow due to the increase in the average age of the general population and the frequency of risk factors associated. Determination of risk factors for onset of knee OA may help in its prevention.^2^

In patients with knee pain, attribution of pain to knee OA should be considered with caution. Because a proportion of knee OA is asymptomatic, in a number of patients, identification of knee OA is not possible due to low sensitivity of radiographic examination. Moreover, in elderly subjects, quadriceps strength, knee pain, and age are more important determinants of functional impairment than the severity of knee OA as assessed radiographically. Available treatments ranging from chondrocyte transplantation to new oral antiinflammatory medications, health education, and further strategies designed to optimize muscle strength may have the potential to reduce a vast burden of disability, dependency, and cost.^3^

Previously highlighted aspects become relevant if we consider the health spending related to OA problems, especially for knee or hip surgeries. Cost‐utility analyses of total hip and knee replacement have been performed by Jenkins et al.^4^ To reduce wastes, decision makers of many health care organizations are regularly faced with different choices regarding the adoption of new technologies, the application of management excellence oriented models and the introduction of quality improvement programmes or process redesign techniques.^5^, ^6^, ^7^, ^8^, ^9^, ^10^, ^11^, ^12^, ^13^, ^14^, ^15^, ^16^

Among the most widespread solutions to minimize cost and improve products and services quality, Lean Six Sigma (LSS), thanks to the synergy of both Lean and Six Sigma methodologies, seems to be one of the most innovative and effective approaches in “Operational Excellence.”^17^ Lean Six Sigma is a combination of lean thinking and Six Sigma aimed at the continuous improvement of a production process through the push for speed and flexibility given by lean thinking and statistical support provided by Six Sigma.

Lean thinking has been used to describe the Toyota Production System, whereas Six Sigma was created in 1987 by Motorola Corporation to improve product quality by identifying errors and mistakes in manufacturing and business processes. The Institute for Healthcare Improvement^18^ affirms that it is possible to apply Lean principles in the context of health care and that the same gains should be created, in efficiency and quality, seen in other areas. In the field of health care, LSS has been used to address numerous health care problems.^19^, ^20^, ^21^, ^22^, ^23^, ^24^, ^25^, ^26^

According to the national and international literature,^27^, ^28^, ^29^, ^30^, ^31^, ^32^ one of the most important indicators to measure the performance of a health care process is the length of hospital stay (LOS) since being in some cases influenced by several factors especially related to an inappropriate organization of the process of care. In fact, excessive LOS is in most cases associated with the lack of standardization of the health care process, generating an unjustified variability from the original LOS.^19^

In Italy, the prevalence of symptomatic OA in people over 60 is 29.9% for the knee, 14.9% for the hand, and 7.5% for the hip.^33^ Given its higher percentage, we will deepen the study on the knee. The knee is the most important location for each of the high disability associated with it and the frequent use of costly surgical replacement. In Italy, the number of interventions on the knee is over 60,000 for year, and every year recording an average increase of 8.9%,^34^ with a related health spending that approaches hundreds millions of euro for hospitalizations and surgeries. In particular, in Campania, the number of operations recorded in the year 2012 was about 3000, rising steadily in the last few years.^34^ Due to the costs imposed on health care institutions, identification of strategies to improve the quality of care provided and, at the same time, contain costs are very important for hospitals.

This study, conducted at the University Hospital “Federico II” of Naples, is a part of a bigger project, entitled “*Application of Lean Six Sigma tools to Orthopedic Surgery*,” about the application of tools of the LSS methodology to improve health care processes. A previously published work regarding the hip replacement surgery has shown very promising results. Particularly encouraged from results of a previous study concerning a LSS application to manage patients undergoing prosthetic hip replacement,^19^ the main objective of this work is to show LSS efficiency and efficacy of LSS and DMAIC approach in developing a clinical pathway, which allows improving quality and reducing costs related to the process of prosthetic knee replacement surgery.

## 2 METHODS

In compliance with a typical LSS improvement process, the DMAIC (*Define*, *Measure*, *Analyse*, *Improve*, and *Control*) roadmap has been adopted to perform the study, as reported elsewhere by the authors.^19^

**Figure 1:**
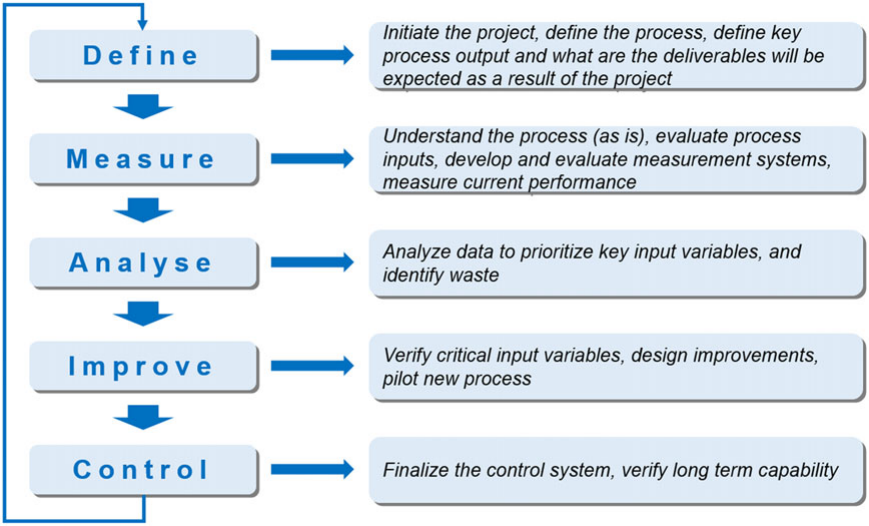
Brief description of the typical lean Six Sigma DMAIC (Define, Measure, Analyze, Improve and Control) phases

The acronym DMAIC is often used to represent the 5 stages of LSS methodology. Many of the steps are identical to those used in the Deming cycle (*Plan‐Do‐Check‐Act*) or in the Lean manufacturing, including definition of the project, metrics to measure various parts of a process, and the process improvement itself. The difference introduced with the Six Sigma, compared with other methods, is the use of statistics to perform data analysis. A synthetic and generic description of the DMAIC phases is shown in Figure 1.

Consistently with the authors' previous work,^19^ the project was developed at the Complex Operative Unit of Orthopaedics and Traumatology of the University Hospital “Federico II,” which has 18 beds dedicated to regular admissions, 6 beds for day surgery activities, and 3 operating rooms.

Data for all the patients involved in the present study were collected from printed medical records and digital information system database of the University Hospital “Federico II” and included anamnestic (age and gender) and clinical variables (dates of admission, surgery and discharge, comorbidities, American Society of Anesthesiologists [ASA] scores). Statistical analyses, including Shapiro‐Wilk and Mann‐Whitney *U* test, were carried out by means of IBM SPSS Statistics 20 software.

A research checklist was also included for this study according to the Standards for Quality Improvement Reporting Excellence guidelines (see research checklist for further details).

### 2.1 Define

The study was conducted by a multidisciplinary team led by the director of the Department of Public Health of the University Hospital “Federico II.” At first, it was developed a *project chart* to define the problem to be solved.

The chart allows visualization of the following:

- title of the project: Lean Six Sigma for the Management of the patient submitted to prosthetic knee replacement surgery;
- problem to be solved: inappropriate prolongation of hospital stay;
- project team members;
- critical to quality (CTQ): LOS; and
- project target: realize corrective measure to reduce the CTQ.

**Figure 2:**
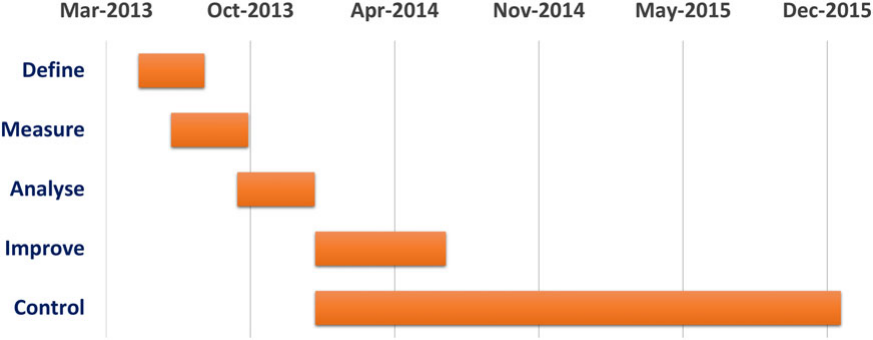
Gantt diagram

In addition, a Gantt diagram was realized to specify the project timetable (Figure 2).

Finally, an *input process output analysis* 35 was carried out for clarifying the following main process characteristics:

- input: surgical device‐medical services;
- process: care process; and
- output: recovery of the functional state of the knee‐diagnostic and therapeutic information‐health.

The LOS, measured in days, was defined as the CTQ of the process. After a literature survey and a discussion with the multidisciplinary team, because the average LOS resulted from the gathered data was longer than 14 days, the objective of the project was determined as the reduction of hospital days less than 14 days.

### 2.2 Measure

During the Define phase, the multidisciplinary team identified the main different characteristics of the project, such as the problem to be solved, the CTQ, and the methods to adopt, whereas during the Measure phase, measurements were carried out to evaluate the performances of the current process. A retrospective data analysis was necessary to make a Value Stream Map of the current process performance.

In this phase, the dataset was obtained from a sample constituted by 148 patients undergoing prosthetic knee replacement surgery (retrospective analysis from June 2011 to May 2013). Among them, 4 patients who had postoperative complications were excluded from the analysis. After the implementation of the clinical pathway (January 2014 to December 2015), information were collected from a sample of 97 patients to establish the effects of the improvement actions on the LOS.

For each patient, the following information was collected:

- gender and age;
- presence of allergies, cardiovascular diseases, and diabetes;
- ASA score;
- prehospitalization;
- date of admission;
- date of surgery; and
- date of discharge.

A systematic chart of the collected data was realized to better understand all the available information.

In addition, statistical analyses (whose results will be presented in the following paragraphs) were carried out to estimate the LOS mean of the sample and to achieve an in‐depth characterization of the chosen CTQ.

To test the normality distribution of data and to carry out further statistical tests, a Shapiro‐Wilk test (*α* = 0.05) was first performed. The assessment of the normality of sampling distribution was necessary to permit further analyses.

A *run chart* and *run tests*, with a significance level *α* of 0.05, was employed afterwards. It allowed us to assess the influence of possible factors affecting the process, such as specific periods of inefficiency in the performance of the process (please refer to section 3 for further details on the run chart).

### 2.3 Analyse

**Figure 3:**
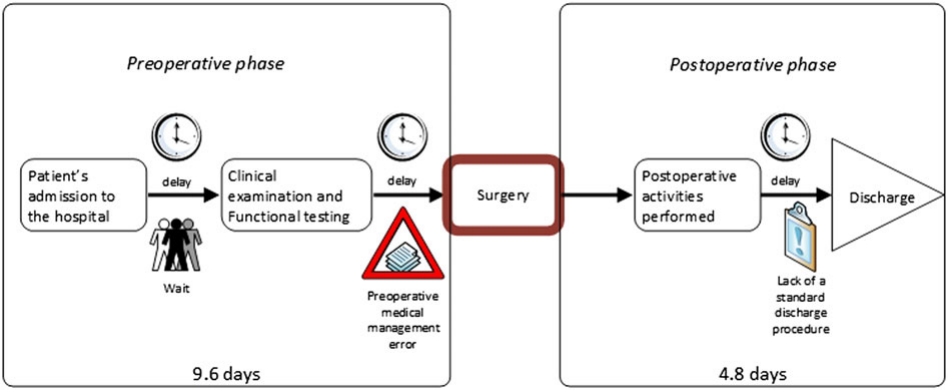
Value Stream Map of the care process for patient undergoing prosthetic knee replacement

On the basis of the achievements of both the Define and Measure phases, the collected data were analysed for recognizing any factor causing process variations. In particular, through the *Value Stream Map*, presented in Figure 3, it was possible to synthetically describe the “as‐is” process, isolating main activities and identifying sources of wastes, delays, and inefficiencies.

Then, an in‐depth understanding of which patient‐related factors (gender, age, allergies, cardiovascular disease and diabetes, and ASA score) could influence the LOS was obtained by means of a statistical analysis. To this aim, a Shapiro‐Wilk test was first performed to assess data distribution for each group. Due to the nonnormality of almost all the examined groups, differences between them were assessed by a Mann‐Whitney *U* test. For those groups who were not dichotomous (age), analysis of variance was carried out. The results of the statistical analysis are reported in section 3.

The last step of the analysis phase has included the following:

1. the administration of a questionnaire (see Appendix A in the supporting information for details about the questionnaire) to orthopaedists, nurses, physical therapists, anaesthesiologists, and consultant physicians of the department to recognize the main causes of inefficiencies in the surgery process; and
2. a brainstorming session aiming at identifying all possible factors related to process inefficiencies. Nurses, physical therapists, and anaesthesiologists of the department and multidisciplinary team members were involved in the brainstorming session.

At the end of the analysis phase, 7 crucial factors have emerged:

- preconceptions against the use of the health care information system;
- wrong preoperative planning;
- lack of standard discharge procedure;
- complex bureaucratic procedures;
- waits for specialist consultancy;
- postoperative complications; and
- waits for clinical examination and functional testing.

Solutions to the emerged problems were reached afterwards.^8^

### 2.4 Improve

Appropriate corrective actions were planned after the Analysis phase. Thanks to the process evaluation obtained by means of the *Value Stream Map* and the brainstorming session, it emerged that one of the most critic weaknesses was linked to the surgery preparation and the preoperative examinations and tests. Based on these observations and in accordance with lean thinking, the team decided to implement a service of prehospitalization as described elsewhere by the authors.^19^

Moreover, as reported on a previous work of the authors,^19^ simplification of complex bureaucratic procedures, standardization of the patient discharge process, and promotion of the health care information system through meetings and information activities for the clinical staff were adopted to optimize the main procedures of the care process, reducing waists and delays.

Starting from January 2014, after implementing the proposed solutions, the length of the hospital stay was monitored. Due to the improvement actions, the team registered a mean LOS equal to 8.3 days, which represents a noteworthy reduction (about 42%) with respect to the 14.2 days calculated before the *improve* phase. Simultaneously, an equally significant reduction (~56%) was obtained for the LOS standard deviation, which changed from 5.2 to 2.3 days.

### 2.5 Control

During the *control* phase, the multidisciplinary team intended to verify the validity of the new clinical pathway developed and then plan strategies to monitor the achieved results over time.

Mann‐Whitney *U* test (significance level *α* of 0.05) was employed to compare LOS between patients operated before and after the project. This comparative analysis was carried out by grouping patients according to the gathered clinical information that were considered in the study (see Table 1). In addition, the percentage of increment in LOS was calculated and reported in Table 1.

**Table 1:** Differences in length of stay (LOS) before and after implementation of the improvement actions

Variable		LOS (Mean ± SD) June 2011 to May 2013	LOS (Mean ± SD) January 2014 to December 2015	LOS Percentage of Increment (%)	*P* value (Mann‐Whitney *U* test)
All patients		14.22 ± 5.17	8.28 ± 2.30	42	<.001
Gender	Male	12.55 ± 4.36	7.00 ± 1.51	44	<.001
	Female	14.81 ± 5.34	8.83 ± 2.38	40	<.001
Age [years]	<60	13.53 ± 4.32	8.22 ± 2.41	39	<.001
	60‐75	13.23 ± 4.40	7.95 ± 2.09	40	<.001
	>75	15.83 ± 6.29	8.85 ± 2.51	44	<.001
Allergies	Yes	13.88 ± 4.78	8.00 ± 2.18	42	<.001
	No	14.45 ± 5.46	8.45 ± 2.39	42	<.001
Cardiovascular diseases	Yes	16.19 ± 5.77	8.32 ± 2.48	49	<.001
	No	12.21 ± 3.54	8.23 ± 2.11	33	<.001
Diabetes	Yes	13.50 ± 4.39	9.27 ± 2.86	31	<.001
	No	14.37 ± 5.33	8.00 ± 2.07	44	<.001
ASA score	I‐II	13.19 ± 4.36	8.32 ± 1.99	37	<.001
	III‐IV	16.44 ± 6.11	8.19 ± 2.92	50	<.001

Regarding the actions planned to ensure result sustainability in the long run, the team, also on the basis of their previous study,^19^ decided for the following:

- periodical review meetings to evaluate the status of the process implementation;
- internal auditing to verify the implemented solutions; and
- periodical updates of the run chart for taking immediate corrective actions.

## 3 RESULTS

Length of stay values before and after the project are reported in Table 1. An average LOS of 14.2 days, with a standard deviation of 5.2, has been registered for patients who have undergone knee surgery from June 2011 to December 2012. This result refers to the “as‐is” process. Furthermore, our statistical analysis shows that the presence of cardiovascular disease along with anticoagulation therapy and the ASA score affect significantly the LOS. These results are analogous to those obtained in the previous work of the authors.^19^ The influence of these factors on the duration of hospitalization was primarily due to waiting for the execution time of the examinations after booking.

**Figure 4:**
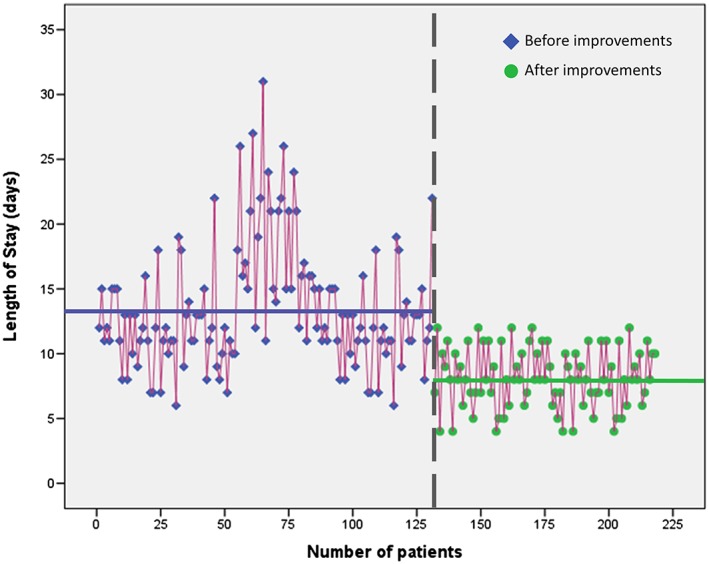
Length of hospital stay (LOS) run chart before (left) and after (right) the improvement actions

Figure 4 displays a complete and easy run chart to monitor the change of the CTQ for both periods (before and after improvements).

Table 1 along with Figure 4 show that the implemented actions of improvement have led to a substantial decrease of LOS, which has been reduced by 42% from a mean value of 14.2 days to 8.3. A simultaneous reduction of 56% has been registered for its standard deviation, which changed from 5.2 to 2.3 days. In particular, for patients with cardiovascular disease or with an ASA score ranging between 3 and 4, there has been a more significant reduction of the duration of hospitalization of about 50%. Moreover, statistically significant differences have been found comparing patients before and after the improvements grouped according to the clinical and demographic variables. Analogous comparisons for further variables (smoke and anticoagulation therapy) are not present in Table 1 because it was not possible to gather the related data.

In comparison with our previous work on the hip replacement surgery, the results here presented are equally promising. Table 2 shows a comparison between the 2 works.

**Table 2:** Comparison between the 2 fundamental studies of the project

Application of Lean Six Sigma Tools to Orthopaedic Surgery		
	First Study	Second Study
Type of intervention	Hip replacement surgery	Knee replacement surgery
# patients before improvements	79	131
# patients after improvements	48	87
Analysis period before improvements	Jun 2011 to Dec 2012	Jun 2011 to May 2013
Analysis period after improvements	Jan 2013 to Dec 2013	Jan 2014 to Dec 2015
Define phase	•Project chart	•Project chart
	•SIPOC diagram	•Gantt chart
		•Input process output (IPO) diagram
Measure phase	•Run chart	•Shapiro‐Wilk and statistical tests
	•Run tests	•Run chart
		•Run tests
Analysis phase	•Value Stream Map	•Value Stream Map
	•Brainstorming	•Brainstorming
	•Ishikawa fishbone	•Interview with health care professional
Improve phase	•Activation pre‐hospital service	•Simplification of complex bureaucratic procedures
	•Simplification of complex bureaucratic procedures	•Standardization of the patient discharge process
	•Standardization of the patient discharge process	•Promotion of the health care information system through meetings
		•Information activities for the clinical staff
Control phase	•Student's *t* test for comparative analysis	•Mann‐Whitney *U* test for comparative analysis
	•Periodical review meetings	•Periodical review meetings
	•Internal auditing	•Internal auditing
	•Periodical updates of the run chart	•Periodical updates of the run chart
Length of stay reduction (%)	44	42

## 4 DISCUSSION

The proposed application of the DMAIC method, which aimed at improving the management of patients undergoing surgery for prosthetic knee, confirms the achievements highlighted in our previous work.^19^

From the obtained results, it has been shown that age and clinical factors of different nature, such as the presence of certain diseases, are related to prolonged LOS. In addition, the average LOS of patients with knee fracture and its standard deviation are reduced by about 42% and 56%, respectively.

Thanks to the multidisciplinary team conducting the project, the DMAIC has been demonstrated to be a highly effective cost reduction strategy for the development and optimization of clinical pathways in the shortest possible time. In fact, taking into consideration the average cost of 1 day of hospital stay at the national level, which is around 674 euro,^36^ our project results in annual cost savings of more than 260,000 euro, as elsewhere highlighted by the authors.^19^ In addition, it offers different tools for the evaluation of critical steps of the process.

For completeness, as reported in Table 2, the comparison between the 2 fundamental studies under the project “*Application of Lean Six Sigma tools to Orthopedic Surgery*” proved the effectiveness of the employed methodology, ie, LSS and DMAIC cycle, both in the hip replacement and in the knee replacement surgery processes. The DMAIC approach, derived from the LSS approach, is a very versatile tool, which is capable of ensuring improvements in health services in both effectiveness and efficiency.

## Supporting information

Supporting info item

Supporting info item
