# Supplementary material for: Improving performances of the knee replacement surgery process by applying DMAIC principles
Source: J Eval Clin Pract. 2017 Sep 26;23(6):1401–7. doi: 10.1111/jep.12810 (PMC6585639; doi:10.1111/jep.12810)
Supplement: Supplementary file 1 — Supporting info item [file JEP-23-1401-s001.docx]

**RESEARCH CHECKLIST^[[1]](#footnote-1)^**

| **Title and Abstract** | |
| --- | --- |
| *Title* | Improving performances of the knee replacement surgery process by applying DMAIC principles |
| *Abstract* | Background: In Italy the number of interventions on the knee approaches hundreds millions of euro for hospitalizations and surgeries. Treatments, technologies and management strategies could help to reduce health spending.  Purposes: Applying process redesign techniques to improve quality and reduce costs related to the process of prosthetic knee replacement surgery.  Methodology: Lean Six Sigma methodology has been employed to decrease the length of hospital stay for patient undergoing prosthetic knee replacement.  Findings: The adopted actions of improvement reduced the length of hospital stay from a mean value of 14.2 to 8.3 days with significant reduction of the average costs related to hospital stay. |
| **Introduction** | |
| *Problem description* | Decision makers of many healthcare organizations are regularly faced with different choices regarding the adoption of new technologies, management excellence oriented models, quality improvement programs and process redesigns techniques to reduce wastes. In Italy, health spending for interventions on the knee approaches hundreds millions of euro for hospitalizations and surgeries. Therefore, identification of strategies to improve the quality of care provided and, at the same time, contain costs are very important for hospitals. |
| *Available knowledge* | Process improvement can be achieved through a mathematical model, by the development of collaborative applications and adoption of ontological relations. Among the most widespread solutions to minimize cost and improve service quality, *Lean Six Sigma* (LSS) seems to be one of the most innovative and effective approach in terms of "Operational Excellence". |
| *Rationale* | The Institute for Healthcare Improvement states that it is possible to apply Lean principles to the healthcare. It has been used to address numerous problems including decreasing length of stay, reducing medication errors, and improving the admissions process. Among the most useful healthcare performances indicators, the length of hospital stay plays a fundamental role. It can be related to an inappropriate organization and/or to the lack of standardization of the healthcare process. |
| *Specific aims* | This work aims at developing an appropriate strategy to reach a significant healthcare cost saving by redesigning the process of prosthetic knee replacement surgery using the Lean Six Sigma methodology. In particular, the objective project is to reduce the length of hospital stay for patients undergoing knee replacement. |
| **Methods** | |
| *Context* | The project was developed at the U.O.C. (Complex Operative Unit) of Orthopedics and Traumatology of the University Hospital “Federico II”. In compliance with a typical Lean Six Sigma improvement process, the *DMAIC* (*Define, Measure, Analyse, Improve, and Control*) roadmap has been adopted to perform the study. |
| *Intervention(s)* | The research was conducted by a multidisciplinary team composed by an orthopedic and trauma surgeon with years of experience, three engineers and one orthopedic surgeon, with experience in health management or in the type of surgery considered.  After an in-depth understanding of the problem achieved through process mapping, data measures and brainstorming activities, in order to optimize the main procedures of the care process, reducing waists and delays, the team decided to:   - Implement a service of pre-hospitalization; - Simplify complex bureaucratic procedures; - Standardize the patient discharge process; - Promote the healthcare information system through meetings and information activities for the clinical staff. |
| *Study of the intervention(s)* | A comparative analysis was carried out by means of the *U Mann Withney test* to compare the Hospital Length of Stay values between patients operated before and after the implementation of the project.  Furthermore, other actions have been planned:   - Periodical review meetings to evaluate the status of the process implementation; - Internal auditing to verify the implemented solutions. |
| *Measures* | Statistical analyses were performed on data collected from printed medical records and digital information system database of the University Hospital "Federico II", which included anamnestic (age and gender) and clinical (dates of admission, surgery and discharge, comorbidities, American Society of Anesthesiologists scores) information about all the patients involved in the study. |
| **Results** | |
| *Results* | The project allowed the identification of variables inﬂuencing the inappropriate prolongation of the length of hospital stay for inpatient treatment (primarily due to waiting for the execution time of the examinations after booking) and the implementation of corrective actions to improve the effectiveness and efﬁciency of the process of care. In fact, the adopted actions of improvement reduced the length of stay by 42%, from a mean value of 14.2 to 8.3 days. A simultaneous reduction of 56% has been registered for its standard deviation, which changed from 5.2 to 2.3 days. |
| **Discussion** | |
| *Summary* | A significant reduction of the average costs of hospital stay can be achieved through the implementation of the proposed strategy. Such a versatile approach could be applied to redesign and improve a wide range of healthcare processes. |
| *Limitations* | Limitations in this study are the relatively small size of the sample and the possible influence of contextual factors such as the Italian Healthcare System. |
| *Conclusions* | We demonstrated that the Lean Six Sigma method is an instrument capable of ensuring an improvement of health services in terms of effectiveness and efficiency. It is also an excellent cost reduction strategy for the development of a clinical pathway in the shortest possible time. Furthermore, the proposed approach is widely applicable as a basic framework for future developments. |

1. *Revised Standards for Quality Improvement Reporting Excellence (SQUIRE 2.0)*

   *Ogrinc G, Davies L, Goodman D et al. Standards for Quality Improvement Reporting Excellence 2.0: revised publication guidelines from a detailed consensus process. J Surg Res 2016;200(2):676-682.* [↑](#footnote-ref-1)
