# Supplementary material for: Improving performances of the knee replacement surgery process by applying DMAIC principles
Source: J Eval Clin Pract. 2017 Sep 26;23(6):1401–7. doi: 10.1111/jep.12810 (PMC6585639; doi:10.1111/jep.12810)
Supplement: Supplementary file 2 — Supporting info item [file JEP-23-1401-s002.docx]

# Improving performances of the knee replacement surgery process by applying DMAIC principles

Giovanni Improta, Giovanni Balato^,^ Maria Romano, Alfonso Maria Ponsiglione, Eliana Raiola, Mario Alessandro Russo, Patrizia Cuccaro, Liberatina Carmela Santillo and Mario Cesarelli

**APPENDIX A**

**INTERVIEW WITH THE MULTIDISCIPLINARY TEAM MEMBERS**

*(orthopaedists, nurses, physical therapists, anesthesiologists and consultant physicians)*

**Name _______________________ Surname _____________________**

**Current position ___________________________________________**

**Years of employment in the current position ____________________**

1. In your opinion, are the main structural requirements (*e.g. beds dedicated to regular admissions, beds for Day Surgery activities, operating room*) in order to provide an efficient and effective performance respected?

___________________________________________________________

___________________________________________________________

___________________________________________________________

1. In your opinion, with regard to available human resources, are all the activities during pre-, peri-, intra- and post-operative stages organized properly?

___________________________________________________________

___________________________________________________________

___________________________________________________________

1. In your opinion, are the multidisciplinary team professionals properly trained and updated regarding the appropriate care process?

___________________________________________________________

___________________________________________________________

___________________________________________________________

1. According to your experience, are protocols or written procedures (*e.g. pre-hospitalization, admission, surgery, discharge*) for an efficient and effective performance widespread in the department?

___________________________________________________________

___________________________________________________________

___________________________________________________________

1. According to your experience, are there optimum ratios between surgical area and other care services (*e.g. consultant, physical therapists*)?

___________________________________________________________

___________________________________________________________

___________________________________________________________

1. According to your experience, is the length of hospital stay shortest possible?

___________________________________________________________

___________________________________________________________

___________________________________________________________

1. In your opinion, does the multidisciplinary team follow the recommended practices for the knee replacement during pre-, peri-, intra- and post-operative stages?

___________________________________________________________

___________________________________________________________

___________________________________________________________

1. **In conclusion,** can you describe the potential of inappropriateness related to both structural and organizational aspects of department and to professional behaviours of the multidisciplinary team members?

___________________________________________________________

___________________________________________________________

___________________________________________________________

1. Also, can you indicate the main reasons (*e.g. management errors, mistakes and lapses, lack of standard procedures, excessive bureaucracy*) for the found problems?

___________________________________________________________

___________________________________________________________

___________________________________________________________
